# Growth and location of bacterial colonies within dairy foods using microscopy techniques: a review

**DOI:** 10.3389/fmicb.2015.00099

**Published:** 2015-02-18

**Authors:** Cian D. Hickey, Jeremiah J. Sheehan, Martin G. Wilkinson, Mark A. E. Auty

**Affiliations:** ^1^Teagasc Food Research CentreFermoy, Ireland; ^2^University of LimerickLimerick, Ireland

**Keywords:** lactic acid bacteria, milk fermentation, bacterial location, cheese, microscopy, fat-protein interface

## Abstract

The growth, location, and distribution of bacterial colonies in dairy products are important factors for the ripening and flavor development of cheeses, yogurts, and soured creams. Starter, non-starter, spoilage, and pathogenic bacteria all become entrapped in the developing casein matrix of dairy foods. In order to visualize these bacterial colonies and the environments surrounding them, microscopy techniques are used. The use of various microscopy methods allow for the rapid detection, enumeration, and distribution of starter, non-starter and pathogenic bacteria in dairy foods. Confocal laser scanning microscopy is extensively utilized to identify bacteria location via the use of fluorescent dyes. Further study is needed in relation to the development of micro- gradients and localized ripening parameters in dairy products due to the location of bacteria at the protein–fat interface. Development in the area of bacterial discrimination using microscopy techniques and fluorescent dyes/tags is needed as the benefits of rapidly identifying spoilage/pathogenic bacteria early in product manufacture would be of huge benefit in relation to both safety and financial concerns.

## BACTERIA WITHIN DAIRY PRODUCTS

Bacteria are naturally present and are used extensively across all areas of dairy and food fermentation, either as natural microflora, or as starter cultures added under controlled conditions (Yang et al., 2012). Their fermentative ability, especially that of lactic acid bacteria (LAB) is based on the creation of an acidic environment through the breakdown of carbohydrates such as lactose, maltose, lactulose and sucrose thereby ensuring preservation of food stuffs. Fermented dairy products are often not manufactured under sterile conditions or with sterile milk (unpasteurized) and this can allow non-starter LAB as well as spoilage or pathogenic bacteria access to the fermenting food system (Montville and Matthews, 2005). LAB’s commonly found in dairy products include strains of *Streptococcus*, *Lactococcus, Lactobacilli, Bifidobacteria, Enterococcus,* and *Pediococci.* Within these species there are numerous strain types which can be used in fermentation processes to give specific acidification and flavor profiles to the final product.

Bacteria associated with dairy fermentations can grow over a wide temperature range from 4 to 50°C. Mesophilic bacteria have an optimum growth range of 25–35°C, while thermophilic species have an optimum range of 37–45°C (Johnson and Steele, 2013). The growth of bacterial cells within dairy foods is heavily influenced by parameters such as pH, water activity and salt-in-moisture levels as well as temperature.

The use of starter bacteria is needed in order to acidify the cheese milk before and during dairy food production. These starter bacteria are inoculated into the milk at their optimum growth temperature (described above) and then stored post manufacture at temperatures ranging from 4 to 12°C (depending on the type of product) in order to slow the growth and acidification of these bacteria. Adjunct cultures such as *Propionibacterium* become active via exposure to warmer temperature ranging from 20 to 25°C for a set period of time and are directly involved in the metabolism of lactate to propionic and acetic acid, water, and CO_2_ (Choisy et al., 2000; Hayaloglu and McSweeney, 2014).

### LACTIC ACID BACTERIA

Lactic acid bacteria are the most common and important starter cultures used in fermented dairy products and may originate from the microflora of raw milks (e.g., bovine, ovine, caprine) but more frequently are inoculated intentionally during product manufacture. The initial role of LAB is to control pH of ripening milk and subsequent dairy products via the conversion of naturally occurring lactose found in milk to lactic acid (glycolysis). The rapid reduction (4–8 h) of pH to below 5.3 in cheese or 4.6 in fermented milk products allows for the control of non-starter microflora as only acid-tolerant bacteria can survive in those conditions (Johnson and Steele, 2013). The secondary function of LAB’s in dairy fermentations is flavor development. Intracellular enzymes released by starter and non-starter bacteria during manufacture and ripening are the main contributors to flavor development via the three main biochemical pathways (glycolysis, lipolysis, and proteolysis). The breakdown of caseins is the most important pathway for flavor development in hard and semi- hard type cheese, which LAB contribute heavily to with the formation of small peptides and free amino acids which can then be further converted to form various alcohols, aldehydes, acids, and esters (Smit et al., 2005). Examples of dairy foods produced through LAB fermentations include cheeses, yogurts and sour creams such as crème fraiche. Examples of common starter LAB used in the dairy industry include *Lactococcus lactis* spp. *lactis*, *L. lactis* spp. *cremoris* (Cheddar), *Lactobacillus helveticus*, *Lb. delbruecki* spp. *bulgaricus* and *Lb. casei* (Swiss-/Italian-type cheese), *Streptococcus thermophilus* (Swiss-type cheese/yogurts) and *Lb. acidophilus* (yogurts, soured creams; Leroy and De Vuyst, 2004). These various starter cultures can often be used on their own or as part of a culture mix, incorporating positive aspects from various bacterial strains. Swiss type cheeses display the symbiotic role of mixed starter cultures. Lactose is converted to galactose and L- lactic acid by *S. thermophilus* and *L. helveticus* metabolizes this galactose to L- and D- lactic acid as *S. thermophilus* is incapable of doing so (Fox et al., 1990).

Due to food safety concerns, commercially produced dairy products such as cheeses and yogurts are increasingly manufactured from pasteurized milk. Pasteurization inactivates pathogenic bacteria, but also results in a significant reduction, or inactivation of, naturally occurring microflora populations. Further control is achieved through competitive inhibition. Non-starter lactic acid bacteria (NSLAB) contribute toward flavor development in dairy foods. They are described as adventitious species which, in the case of dairy products, can originate from the factory environment or from the raw milk where they are present as adventitious contaminants (Crow et al., 2001). The most common NSLAB species found in the dairy industry are variants of Lactobacilli (*Lactobacillus casei*, *L. paracasei*, *L. plantarum*, *L. curvatus*, *L. brevis,* and *L. fermentum*) and common non-*Lactobacillus* species include *Pediococcus acidilactici*, *P. pentosaceus*, *Enterococcus durans*, *E. faecalis,* and *E. faecium* (Settanni and Moschetti, 2010).

While LAB account for the majority of bacteria present in dairy foods, either naturally occurring or through deliberate inoculation, other types of bacteria are also used within the dairy industry. These include adjunct cultures, defined as those added to fermented products for reasons other than acid formation (Giraffa, 2003), such as *Staphylococci*, *Micrococci, Enterococci,* and *Propionibacterium* (Chamba and Irlinger, 2004). The latter are used extensively in the manufacture of Swiss- type cheeses in order to produce the distinctive nutty flavor and metabolize lactate to acetate and carbon dioxide (CO_2_) creating the characteristic eyes associated with these types of cheeses. *Propionibacterium* are responsible for high levels of lipolysis associated with Swiss- type cheese and have been shown to have up to 100 times more lipolytic activity than LAB’s (Chamba and Perreard, 2002; Chamba and Irlinger, 2004). Other adjunct culture types are not directly involved in cheese manufacture but become active only during ripening and include yeasts (*Geotrichum candidum*, *Saccharomyces cerevisiae*) and molds (*Penicillium camemberti* and *P. roqueforti*; Chamba and Irlinger, 2004).

### PATHOGENIC AND CONTAMINANT BACTERIA

Bacteria may also be present in dairy foods as undesirable food spoilage or pathogenic agents. These undesirable bacteria may include psychrotophic bacteria (*Pseudomonas fluorescens* and *P. putrefaciens*) *Listeria monocytogenes*, *Salmonella* spp., *Escherichia coli*, *Staphylococcus aureus*, *Clostridium botulinum*, *C. perfringens*, *C. tyrobutyricum,* and *Vibrio cholera* (Giraffa, 2003; Oliver et al., 2005; Gálvez et al., 2008; Machado et al., 2013). Proteases and lipases released by psychrotrophic bacteria in milk such as *Pseudomonas fluorescens* and *P. putrefaciens* can cause bitterness and off flavors in dairy products. These enzymes are heat stable and therefore unaffected by standard pasteurization temperatures (72–74°C for 15–30 s), allowing for the development of off flavors in fermented dairy products (Sheehan, 2013).

The main pathogenic bacteria of concern in the dairy industry are those which are capable of surviving the manufacturing process of cheeses, yogurts and soured creams. *L. monocytogenes* is a gram positive bacterium responsible for causing gastroenteritis along with listeriosis, which in turn can cause serious illness through sepsis or meningitis. The fatality rate from extreme listeriosis ranges from 20 to 30% (WHO, 2004; Carpentier and Cerf, 2011). *E. coli* is a gram negative bacterium which occurs naturally in the lower intestine but certain strains can cause gastroenteritis and urinary tract infections. *E. coli* in dairy products such as yogurts results from post pasteurization contamination. *E. coli* O157 has been shown to be able to survive the acidic conditions associated with yogurt manufacture thus causing serious health risks to consumers (Cirone et al., 2013). *Salmonella* spp. consists of several gram negative species each capable of causing food borne illness. Two of the most common found in dairy foods are *S*. *typhimurium* and *S*. *enteritidis* (Leyer and Johnson, 1992). *S. aureus* is a gram positive bacterium and one of the world’s leading causes of food borne illness, such as gastroenteritis which is caused by the ingestion of enterotoxins produced by the bacteria. Therefore, even if the bacteria are killed via heat or pressure treatment, the heat resistant enterotoxin remains (Gálvez et al., 2008; Fleurot et al., 2014). The acidic environment created by the fermentation of dairy products, high levels of cleanliness and hygiene practice throughout manufacturing plants and correct storage of dairy products help prevent the growth and contamination of products with these undesirable pathogenic and food spoilage bacteria.

### BACTERIOCINS AND PROBIOTICS

Bacteriocins are described as low-molecular mass proteins or peptides produced via bacterial ribosomal synthesis. They have an antimicrobial mode of action usually restricted to related Gram positive bacteria (Caplice and Fitzgerald, 1999; Leroy and De Vuyst, 2004). Bacteriocins are used as a mode of biopreservation. This is the extension of a food’s storage life and increased food safety due the antibacterial products of natural or controlled microflora (Stiles, 1996; Gálvez et al., 2008). Many LAB are bacteriocin producers along with certain species of *Enterococci (E. faecalis* and *E. faecium)* which produce enteriocins (Giraffa, 2003). Examples of two commercially produced bacteriocins are nisin (Nisaplin in commercial form) which is produced by fermentation of milk based substrates by strains of *Lactococcus lactis* and Pediocin PA-1/AcH (ALTA 2341 in commercial form) which is produced from *Pediococcus acidilactici* (Gálvez et al., 2008). Nisin is used extensively throughout the dairy industry to prevent gas blowing in semi-hard and hard type cheeses caused by *C. tyrobutyricum* and as an antimicrobial agent in clotted and heat treated creams preventing the growth of *Bacillus cereus* even at low concentrations and in sliced cheeses it reduces the populations of *Listeria innocula* and *S. aureus* (Scannell et al., 2000; Gálvez et al., 2008).

Probiotics improve the nutritional value of a food and when administered in certain quantities provide health benefits to the host (Granato et al., 2010). Many LAB’s display probiotic characteristics. The most common certified probiotic bacteria strains used in foods today consist of Gram-positive *Lactobacillus* and *Bifidobacterium* species, both of which are found heavily within the human gastrointestinal tract. The afore mentioned bacteria are linked to health benefits such as reduced lactose intolerance, relief from some diarrhoeas, lower blood cholesterol, increased immune response and preventions of certain cancers (Savadogo et al., 2006). The numbers of live bacteria reaching the gut is critical for probiotic efficacy and for this reason, quantifying and visualizing the bacterial colonies in a food prior to ingestion is very important.

### LOCATION OF BACTERIA

The location of bacteria within dairy food matrices is of interest due to the possible effects these bacteria and their enzymes may have on ripening, particularly at the microscopic level in cheese and yogurts. The effect of bacterial colony size, distribution, and where colonies locate may all have an effect on the rate of enzyme release and localized ripening. In order to investigate these possibilities we need to visualize the behavior of developing bacterial colonies using microscopy techniques as discussed below. Bacterial distribution is not homogenous throughout dairy products as all types of bacterial cells (starter, non-starter, spoilage, pathogenic) become entrapped in the developing protein matrix resulting in random distribution of bacterial colonies (Fitzsimons et al., 2001). The immobilization of bacteria therefore requires the diffusion of substrates to the bacterial colony location and resulting metabolites must also diffuse through the protein matrix, resulting in the potential developments of micro-gradients in pH and water activity in and around the bacterial colonies (Floury et al., 2010). Initial studies suggest an interaction between milk fat globules and starter bacterial cells/colonies due to the regular location of bacterial colonies in close proximity or in direct contact with milk fat globules and their membranes (Laloy et al., 1996). Bacteria have since been shown to preferentially locate at the fat–protein interface and sometimes within whey pockets in dairy products.

### IMPORTANCE OF BACTERIA IN DAIRY FERMENTATIONS

The growth of bacteria within fermenting food products is of extreme importance as this growth rate determines final cell numbers, acidification rates and thus the intensity of the fermentation process. Starter bacteria are also responsible for releasing intracellular enzymes upon death and subsequent lysis of the cell membrane. These enzymes catalyze a wide range of metabolic pathways (lipolysis, proteolysis, and glycolysis) which result in the formation of flavor compounds adding to flavor development in many cases of fermented foods (Wilkinson et al., 1994; Wilkinson and Kilcawley, 2005; Steele et al., 2013).

Each bacterial cell is believed to grow and form a colony within the food matrix after inoculation and in certain cases immobilization within the matrix (Jeanson et al., 2011). It is the activity, location, and environment of these colonies which are of interest in this review. To date a lot of research has been undertaken with relation to bacterial growth in various conditions in dairy foods. This review will focus on colony growth, location, and influence on the surrounding environment of starter, non-starter LAB, spoilage and pathogenic bacteria in dairy products such as cheeses, sour creams and yogurts using microscopy to accurately quantify and visualize the bacteria in the food matrices.

## ANALYTICAL TECHNIQUES

Methods for analysis of food can be separated into separate categories such as microbiological, microscopy, sensory, physical, and physico-chemical. These methods can be destructive or non-destructive in their procedure. For this review we will focus on the microscopy methods associated with dairy food analysis. Examples of non-destructive microscopic methods include confocal laser scanning microscopy (CLSM), cryo- and regular scanning electron microscopy (SEM), and transmission electron microscopy (TEM) which have helped to map the location and distribution of bacterial colonies in dairy foods.

Other rapid and reliable methods for enumerating and identifying bacterial cells which include polymerase chain reaction (PCR) amplification and its variants including PCR-denaturing gradient gel electrophoresis (PCR-DGGE) and PCR-temporal temperature gradient gel electrophoresis (PCR-TTGE; Ndoye et al., 2011; Postollec et al., 2011; O’Sullivan et al., 2013). PCR targets a specific sequence such as the 16S rRNA gene in conjunction with genera/species specific primers. The 16S rRNA gene is universal amongst bacteria and large databases exist for specific species of most food related bacteria. They have been used to determine the presence and quantity of undesirable bacteria such as *C. tyrobutyricum* and certain *Lactobacillus* communities in cheese. However, the issue with these methods of bacterial detection is the lack of information we obtain relating to bacterial colony location and distribution within dairy foods. For these reasons, this review focuses on microscopy methods allowing for the visualization of bacterial colony location and distribution.

### CONFOCAL LASER SCANNING MICROSCOPY

The use of CLSM in food analysis has been at the forefront in recent years due to its ability to image individual components within a food matrix via the use of various fluorescent dyes (Auty et al., 2001, 2013; Romeih et al., 2012). This method is favored as a result of its ability to visualize thin optical sections below the surface of a sample due to the laser scanning function. CLSM uses argon and/or helium–neon lasers to individually obtain images from thin sections of sample which can then be stacked together in order to create a three dimensional (3D) image of a without disturbing the internal structure (De Freitas et al., 2007; Ong et al., 2011b; El-Bakry and Sheehan, 2014). Another advantage is its ability to analyze various components simultaneously via the use of fluorescent labels and stains, allowing for fat, protein, and bacterial colony location to be identified from one sample (Ong et al., 2010, 2011a, 2012, 2013b; Abhyankar et al., 2011). Commonly used fluorescent dyes in relation to dairy products and their components are fast green and rhodamine for the protein fractions, nile red for fats, and Oregon green 488/514 for determining localized pH. Bacterial viability is commonly determined using a LIVE/DEAD *Bac*Light viability kit consisting of two fluorescent nucleic acid stains SYTO9 (green) and propridium iodide (PI; red). SYTO9 permeates both viable and non-viable cell membranes, while PI only permeates damaged cell membranes which in turn negate the SYTO9 fluorescence. Thus, viable bacterial cells fluoresce green and those with a damaged or non-viable membrane fluoresce red (Auty et al., 2001). Bacterial location alone can be measured using Acridine orange which is a fluorescent dye which stains the DNA of bacteria (Lopez et al., 2006). This methodology has also been utilized recently to identify and track pathogenic bacterial growth in dairy foods (Fleurot et al., 2014).

### SCANNING ELECTRON MICROSCOPY

Conventional SEM involves the generation of an electron beam which interacts with a given sample resulting in the emission of multiple secondary electrons. The image obtained is based on the electrons which scatter back when the electron beam strikes the surface of the sample (McMullan, 2006). In order to obtain a high number of secondary electrons and therefore give a clear image, a conducting layer is often placed over the sample surface to prevent charging (El-Bakry and Sheehan, 2014). The dehydration of the sample using a series of ethanol concentrations is necessary prior to examination, which must also be carried out under vacuum. This method has been used to study food microstructure for many years, offering a clear concise image of a samples surface showing fat, protein, and bacterial location (Pitino et al., 2012). The limitations associated with this methodology are the labor intensive sample preparation and the ability to only view the topographical area of a sample in addition to artifacts (Tunick et al., 2002).

Cryo-SEM comprises conventional SEM with a cryo-chamber attached allowing for the microscopic examination of dairy foods high in moisture, fat or air, i.e., cheese, yogurts, and soured creams. Operating at temperatures below -80°C, it utilizes liquid nitrogen in order to ultra-freeze samples. The advantages of this method over conventional SEM include a substantially reduced sample preparation time and a greater ability to view fat components which can become distorted due to the dehydration and defatting steps associated with conventional SEM. The use of this method has increased in recent years, in conjunction with techniques such as CLSM, for the study of dairy food microstructure and microbial population (Hassan et al., 2003; Romeih et al., 2012; Martinovic et al., 2013; Ong et al., 2013a).

### TRANSMISSION ELECTRON MICROSCOPY

Transmission electron microscopy is similar to its scanning counterpart in that a beam of electrons are used but in this case the electrons pass through (transmit) the sample and the image generated is based on the scatter of these electrons, therefore samples need to be very thin (0.1–0.2 μm). In relation to dairy analysis, replica type TEM is most commonly used. This involves either resin embedding and ultrathin sectioning or freeze-fractured replicas (Kaláb et al., 1995). Advantages include the best resolution of all electron based microscopy techniques allowing for greater examination of a samples ultra-structure (Laloy et al., 1996) and was first used on cheese by Green et al. (1981). Disadvantages consist of high cost, labor intensive sample preparation and possible presence of artifacts due to the use of osmium tetraoxide, which can cause fat and proteins to be misinterpreted due to inadequate fixing of the structures during sample preparation (Reis and Malcata, 2011; Auty et al., 2013; El-Bakry and Sheehan, 2014).

The use of these microscopy techniques in relation to bacterial location, survival and distribution in cheeses, yogurts and soured creams are discussed below in relation to the various studies which have been conducted on this important topic.

## LOCATION OF BACTERIAL COLONIES IN DAIRY PRODUCTS

Location and distribution of various types of starter bacteria, NSLAB, contamination bacteria, and spoilage bacterial strains in a number of cheese varieties has been widely studied but rarely visualized using microscopic methods. (Hannon et al., 2006; Lopez et al., 2006, 2007; Jeanson et al., 2011). The ability to visualize the location of these bacteria within the developing protein matrix is of huge importance in relation to food quality, consistency and safety.

Jeanson et al. (2011) studied bacterial distribution in a real food matrix (cheese) showing the spatial distribution of bacterial colonies at various levels of inoculation using CLSM. Bacterial colonies were shown to be randomly distributed which fit the proposed Poisson model. Their results supported the theory that increased inoculation levels (10^7^ CFU/g) resulted in smaller colonies and displayed a sevenfold increase in the interfacial area of exchange with the cheese matrix compared to colonies formed at lower inoculation levels (10^4^ CFU/g) as shown previously by McKay et al. (1997). Colonies can consist of bacterial cells in various physiological states of growth and McKay et al. (1997) have previously shown bacterial cells which are in the exponential phase of growth to be located on the colony exterior touching the matrix, most likely resulting in a high level of metabolic activity. This hypothesis allows for the assumption that the larger the interfacial area, the greater the bacterial activity on the food matrix which will in turn influence ripening. At higher levels of inoculation (10^6^ and 10^7^cfu/g) colonies were located extremely close together with mean distances of 25–30 μm between colonies (Jeanson et al., 2011).

**FIGURE 1:**
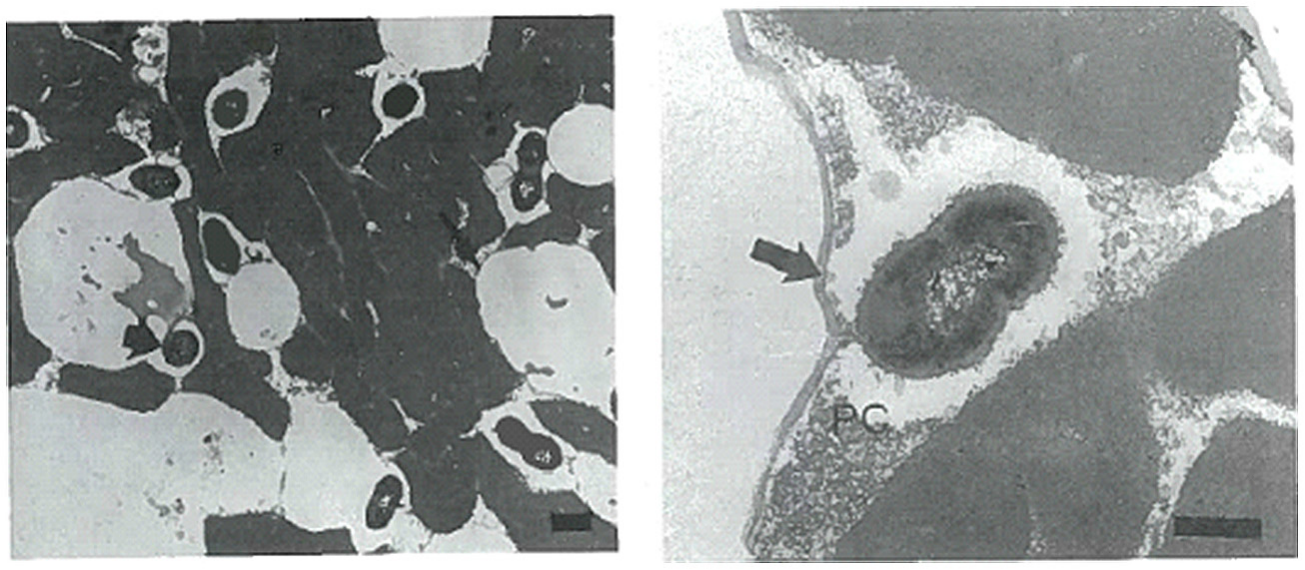
**Transmission electron microscopy (TEM) images (ultrathin sections) of starter bacteria (*Lactococcus lactis* subsp. *cremoris* KB) location in full-fat Cheddar cheese in image 1 (left).** Image 2 (right) shows the starter strain KB locating in close proximity to the MFGM (arrow) together with proteolysed casein protein (PC). The Bar represents 400 nm. Reprinted with permission from Laloy et al. (1996) and Elsevier. Copyright (1996) Elsevier.

Studies have been conducted into the possible location of bacterial micro-colonies within the cheese matrix. Laloy et al. (1996) used TEM to observe the location and distribution of starter bacteria in fat-free, 50% reduced fat and full fat Cheddar cheese (**Figure 1**). They found bacterial populations to be directly related to the fat content of the cheese. Compared to fat- free cheese, starter populations were 30–100% and 4–10 fold higher in 50% reduced fat and full fat cheese, respectively. Bacteria were found to be located in direct contact with the milk fat globular membrane (MFGM) or located at the casein–fat interface. As ripening progressed (>1–2 months) bacteria seemed to become imbedded within or located inside the MFGM itself.

**FIGURE 2:**
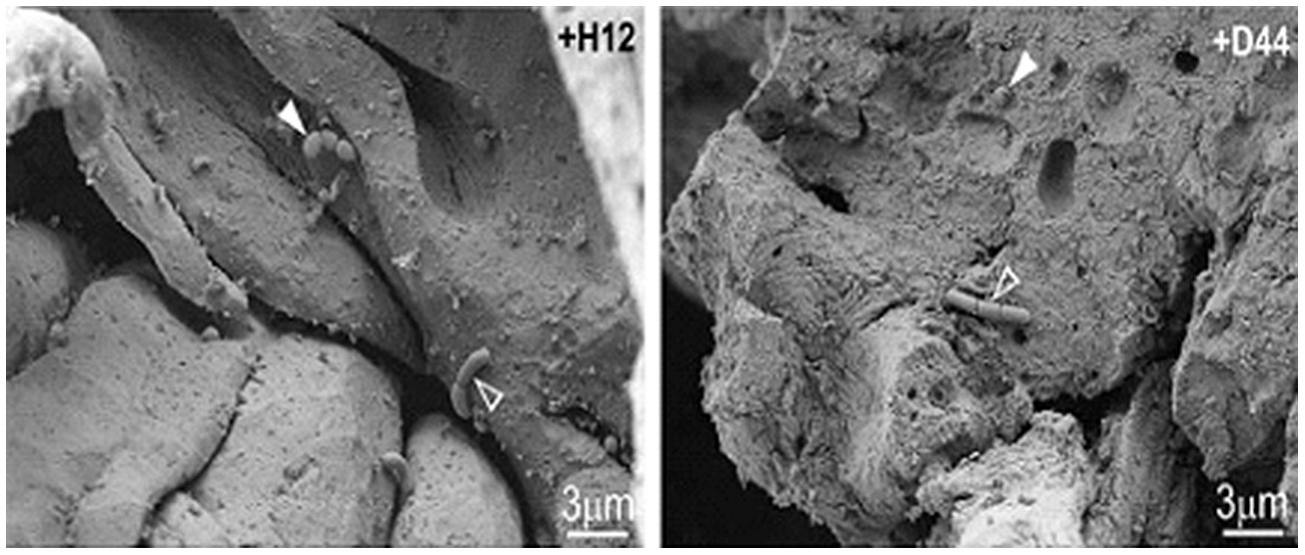
**Scanning electron microscopy images of naturally occurring microflora (arrows) and strains of *Lb. rhamnosus* [+H12 (left) and +D44 (right)] (open arrows) on the surface of cheese after stomacher treatment.** Reprinted with permission from Pitino et al. (2012). Copyright (2012) Elsevier.

Pitino et al. (2012) utilized both SEM and CLSM in order to track the survival of *Lb. rhamnosus* inoculated in cheese during simulated human digestion. They showed the interaction between *Lb. rhamnosus* and the cheese starter bacteria both of which appear to form colonies at the fat–protein interface or in contact with whey pockets (**Figure 2**).

**FIGURE 3:**
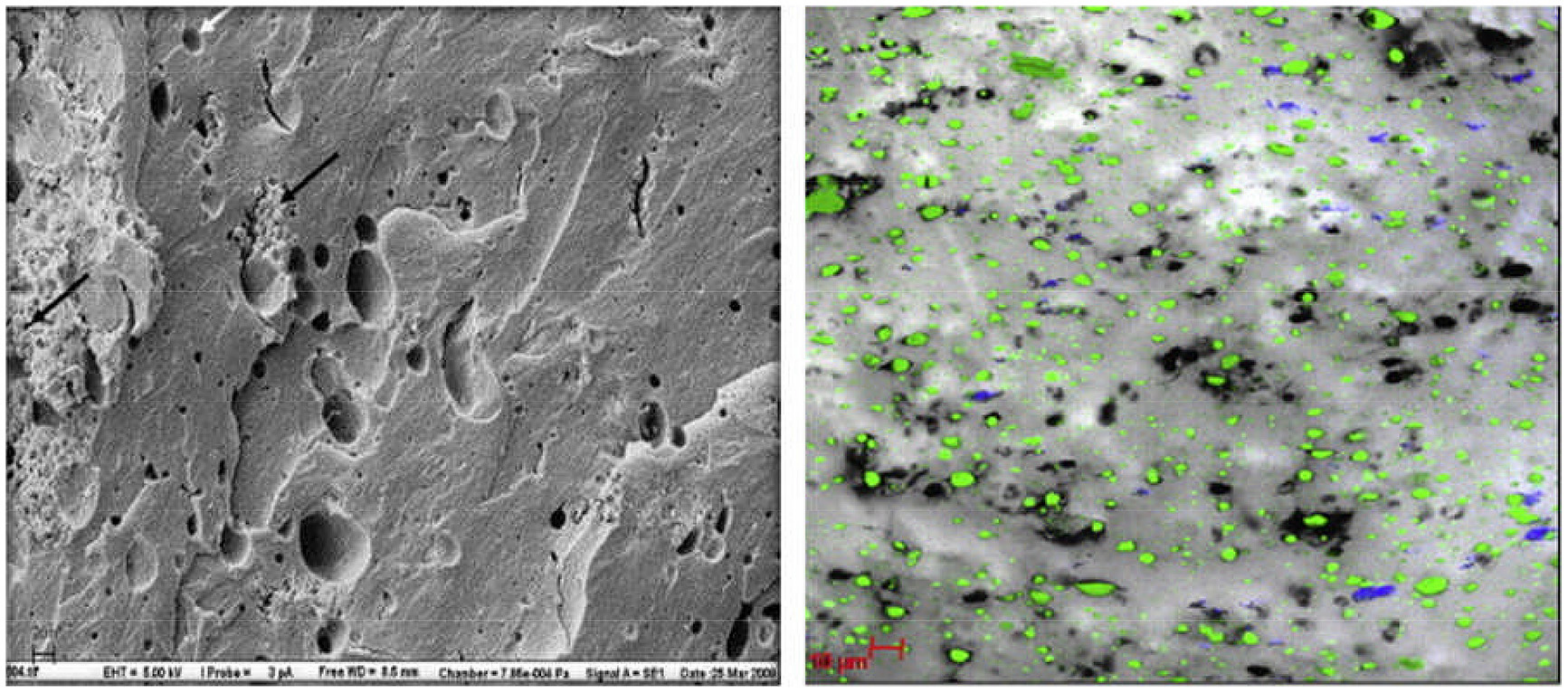
**Scanning electron microscopy (left) image of low-fat cheddar cheese matrix showing colonies of starter cultures (*L. lactis*) (black arrows) and areas previously occupied by fat globules (white arrows); scale bar = 20 μm.** CLSM (right) image of cheese matrix with protein in gray, fat coded in green and bacterial colonies in blue; scale bar = 10 μm. Reprinted with permission from Romeih et al. (2012). Copyright (2012) Elsevier.

**FIGURE 4:**
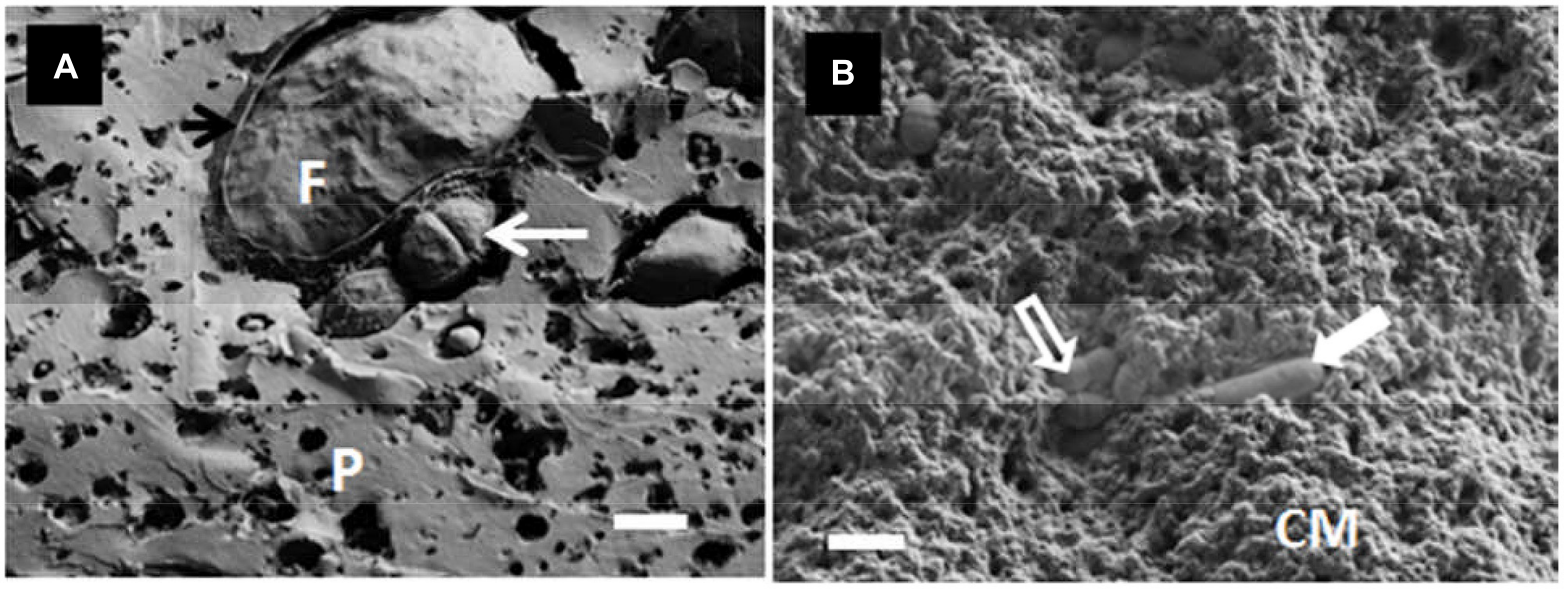
**(A)** Cryo-SEM image of a Cheddar-type cheese showing the location of the starter bacteria (*S. thermophilus*) (white arrow) and fat globule (F), including fractured MFGM (black arrow), within the protein network (P) on day 12 of ripening; scale bar = 1 μm. **(B)** Cryo-SEM image of starter bacteria in yogurt showing both *S. thermophilus* (open arrow) and *Lb. delbruecki* subsp. *bulgaricus* (closed arrow) entrapped within the acidified casein-based matrix (CM); scale bar = 2 μm. Auty, unpublished results.

Romeih et al. (2012) observed the location of *L. lactis* ssp. *lactis* ML8 using SEM in conjunction with CLSM (**Figure 3**), while Ong et al. (2013a) used cryo-SEM to detect the location of a mixture of mesophilic starter bacteria in full-fat Cheddar cheese. Auty (unpublished results) also used the same technique to show exopolysaccharide-producing lactic acid bacteria in Cheddar cheese and starter bacteria in yogurt, respectively, (**Figure 4**). As in the case of many others, the images appear to show preferential location of bacteria at the protein–fat interface. Images produced of yogurt show the entrapment of bacterial cells amongst the acidified casein-based matrix.

**FIGURE 5:**
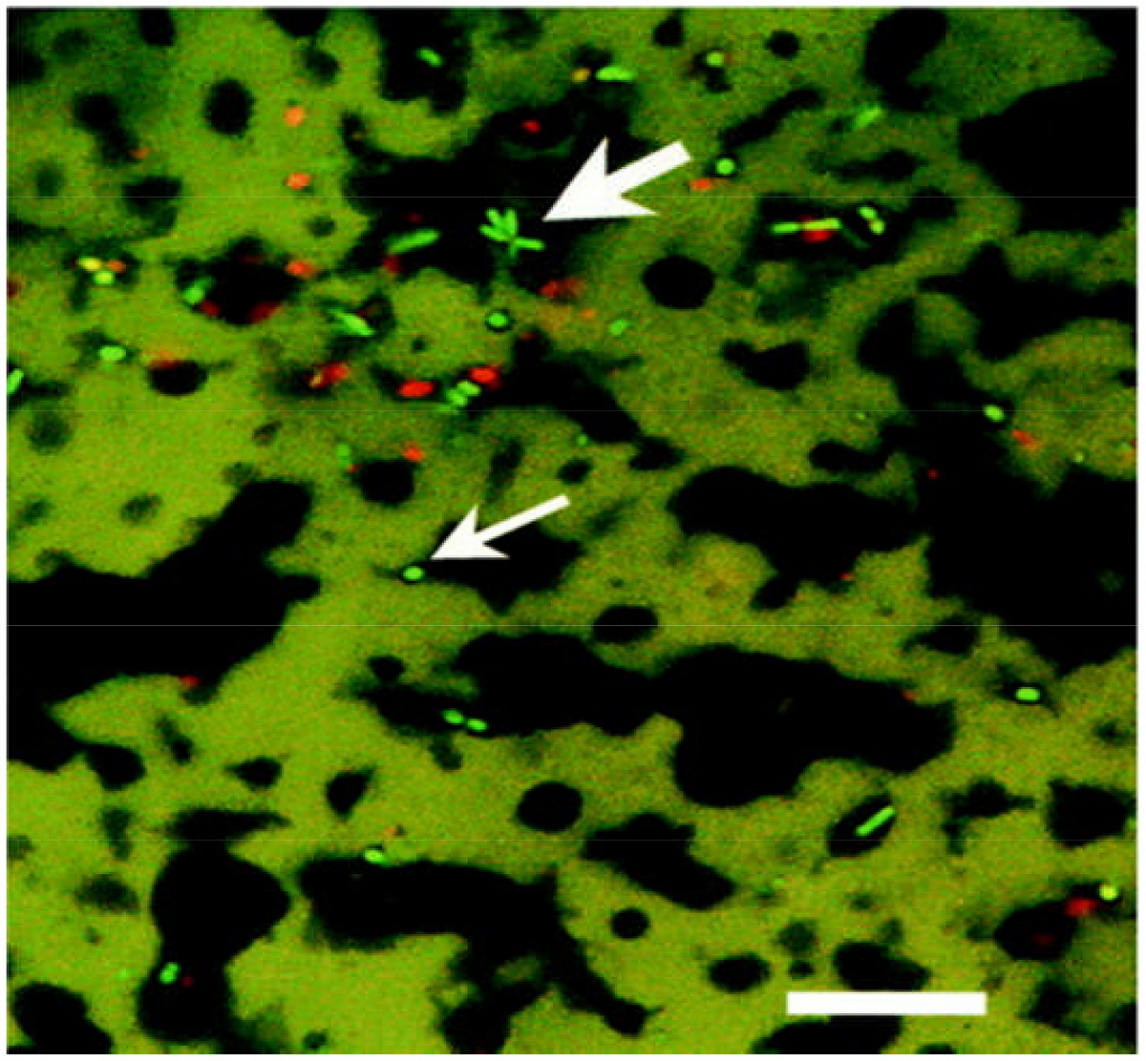
**Confocal laser scanning microscopy image of probiotic cheddar cheese showing star shaped clusters of live (bright green), presumptive Bifidobacteria, (large arrow), and dead (red) bacterial cells at fat (black)/protein (green) interface and presumptive NSLAB bacteria (small arrow).** Scale bar = 25 μm. Reprinted with permission from Auty et al. (2001) and American Society for Microbiology (ASM).

**FIGURE 6:**
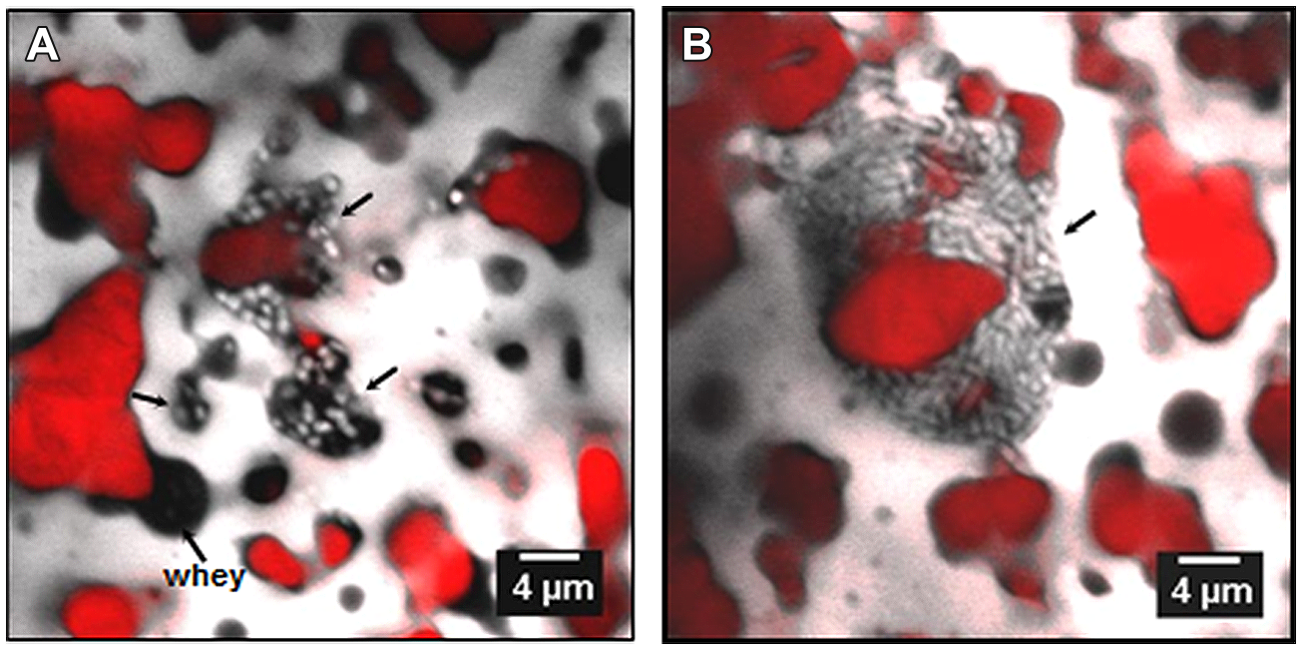
**Confocal laser scanning microscopy images of bacteria in Emmenal cheese after 1 day of ripening, showing location of bacterial colonies (light color) in whey pockets (**A**, black areas) and at the interface **(B)** between fat (red) and protein (gray).** Adapted from Lopez et al. (2006) with permission from the authors.

Auty et al. (2001), Hannon et al. (2006), and Lopez et al. (2006, 2007) used CLSM to visualize bacterial colonies in various cheeses such as Cheddar- type and Swiss- type cheeses made with ultrafiltered milk, respectively. In each case bacterial colonies were observed at the fat–protein interface also (**Figures 5 and 6**, respectively).

**FIGURE 7:**
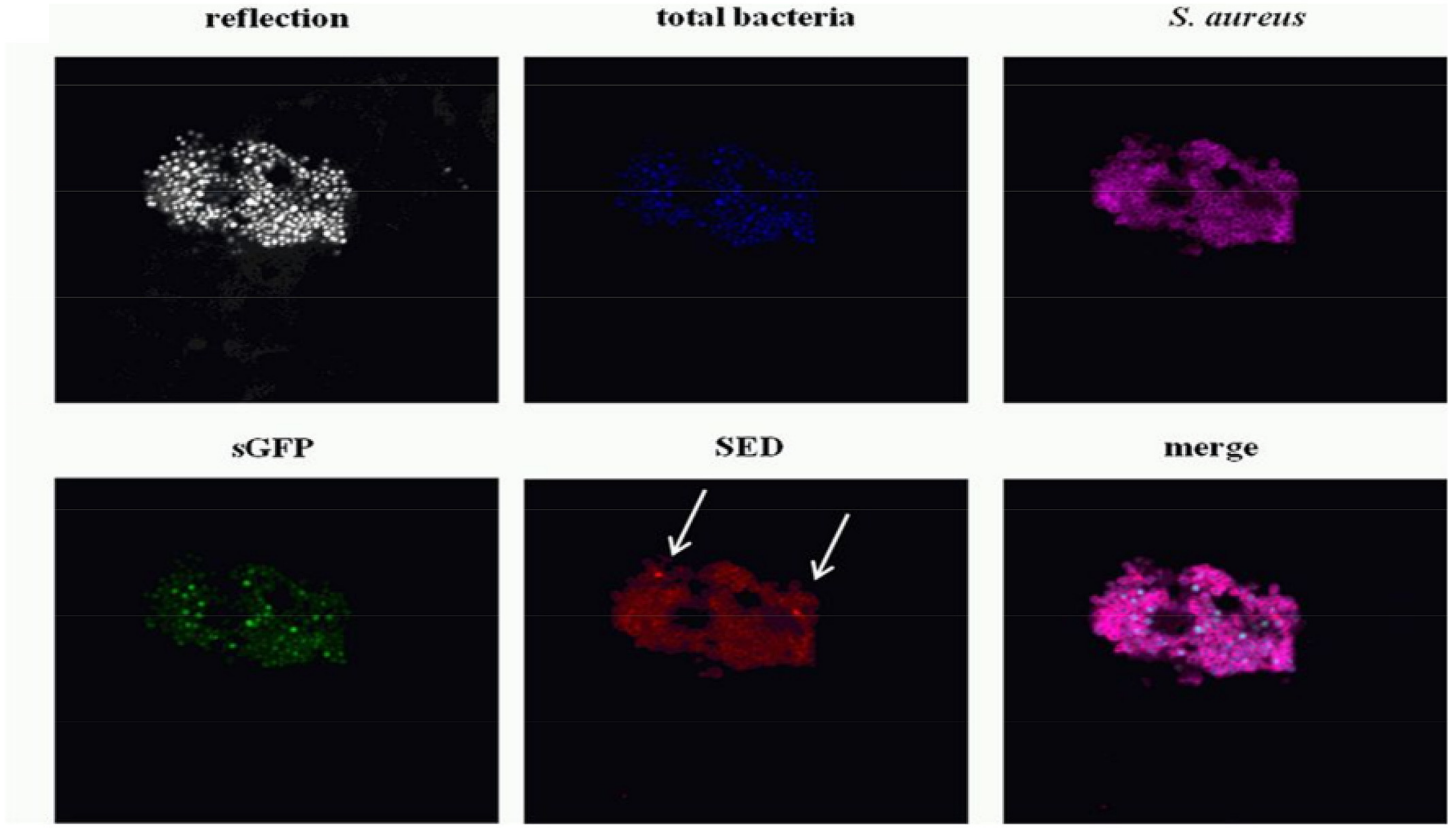
**Confocal laser scanning microscopy images of dairy starter bacteria (Blue), synthetic green fluorescent protein (sGFP) (Green), Staphylococcal enterotoxin D (SED) (Red) and *S. aureus* (magenta) on the cheese surface 14 days into ripening.** The cheese sample structure is visualized by the reflection of the 405-nm laser diode in a grayscale image. Reprinted (adapted) with permission from Fleurot et al. (2014). Copyright (2014) ASM.

Fleurot et al. (2014) employed CLSM in order to locate the food borne pathogenic bacteria *S. aureus* in a cheese system. They found *S. aureus* formed colonies on the exterior surface of the cheese with colonies in the core a rarity, leading to the conclusion that *S. aureus* entrapped in the interior of the cheese did not multiply, while those on the aerated surface continued to multiply and form large colonies (**Figure 7**).

The application of microscopic techniques to investigate bacteria localization in dairy foods has been limited and in cases where it has been used, bacterial location is often observed as a result of investigating components such as fat content. Studies rarely focus on bacterial location and distribution specifically (Lopez et al., 2006, 2007; Romeih et al., 2012; Ong et al., 2013a). Despite many of the aforementioned studies confirming that bacteria in dairy foods invariably locate on or in close proximity to the fat–protein interface or in contact with whey pockets, the effect this localization has on micro-gradients (pH, water activity), flavor development and overall product quality is still relatively unknown. Fleurot et al. (2014) is, to date, the only published work which uses CLSM in conjunction with fluorescent stains in order to discriminate between starter and pathogenic bacteria.

## CONCLUSION

This review provides an overview on current information regarding use of microscopic techniques to investigate the growth and localization of bacteria within dairy based fermented foods. Such studies on bacterial colony location and distribution have not yet addressed the relationship between colony location and on product quality, consistency and on ripening parameters. However, the use of microscopy has made the visualization of bacteria in food matrices possible and allows for the enumeration, location, and distribution of starter LAB, NSLAB, spoilage, and pathogenic bacteria via non-destructive methods. Microscopy methods such as CLSM and cry-SEM allow for rapid sample analysis and CLSM allows for the detection of spoilage and pathogenic bacteria via the use of specific fluorescent dyes. Further study is greatly needed in this area regarding the influence bacterial location at the fat–protein interface has on localized micro-gradients and ripening parameters in a wide variety of dairy products. Microscopy allows for bacteria to be visualized within a solid food system and this tool is key to understanding bacterial behavior and influences on ripening fermented products. The discrimination of minority spoilage or pathogenic bacterial populations from the highly dense starter and non-starter dairy populations is an area which requires immediate attention as the benefits of possible rapid analysis and discrimination would be of great benefit to the entire food industry. Recent developments in super-resolution optical microscopy and confocal Raman microscopy could be used in future to further characterize the microflora of solid foods and the localized biochemical transformations they influence.

## Conflict of Interest Statement

The authors declare that the research was conducted in the absence of any commercial or financial relationships that could be construed as a potential conflict of interest.
